# Importance of Skin Changes in the Differential Diagnosis of Congenital Muscular Dystrophies

**DOI:** 10.1155/2016/3128735

**Published:** 2016-03-31

**Authors:** Uluç Yis, Figen Baydan, Mert Karakaya, Semra Hız Kurul, Sebahattin Cirak

**Affiliations:** ^1^Division of Child Neurology, Department of Pediatrics, School of Medicine, Dokuz Eylül University, 35340 İzmir, Turkey; ^2^İzmir Tepecik Training and Research Hospital, Division of Child Neurology, 35040 İzmir, Turkey; ^3^University Hospital of Cologne, Institute of Human Genetics, 50931 Cologne, Germany; ^4^University Hospital of Cologne, Department of Pediatrics, 50931 Cologne, Germany; ^5^Center for Molecular Medicine, 50931 Cologne, Germany

## Abstract

Megaconial congenital muscular dystrophy (OMIM 602541) is characterized with early-onset hypotonia, muscle wasting, proximal weakness, cardiomyopathy, mildly elevated serum creatine kinase (CK) levels, and mild-to-moderate intellectual disability. We report two siblings in a consanguineous family admitted for psychomotor delay. Physical examination revealed proximal muscle weakness, contractures in the knee of elder sibling, diffuse mild generalized muscle atrophy, and dry skin with ichthyosis together with multiple nummular eczema in both siblings. Serum CK values were elevated up to 500 U/L. For genetic work-up, we performed whole exome sequencing (WES) after Nimblegen enrichment on the Illumina platform. The WES revealed a novel homozygous missense mutation in the Choline Kinase-Beta (*CHKB*) gene c.1031G>A (p.R344Q) in exon 9. Ichthyosis-like skin changes with intense pruritus and nummular eczema may lead to clinical diagnosis in cases with megaconial congenital muscular dystrophy.

## 1. Introduction

The congenital muscular dystrophies (CMD) are a group of diseases that show great clinical and genetic heterogeneity [1]. Megaconial congenital muscular dystrophy (OMIM 602541) is characterized with early-onset hypotonia, muscle wasting, proximal weakness, cardiomyopathy, mildly elevated serum creatine kinase levels, and mild-to-moderate intellectual disability [2]. Muscle biopsy shows dystrophic changes and mitochondrial abnormalities. The center of muscle fibers is usually depleted of mitochondria, but the mitochondria are markedly enlarged at the periphery [3]. The disease is caused by the loss-of-function mutations in choline kinase-beta (*CHKB*). It is suggested that altered phospholipid composition in muscle mitochondrial membrane may lead to mitochondrial structural and functional abnormalities [4]. We report two siblings with megaconial congenital muscular dystrophy and the clinical relevance of skin changes in the differential diagnosis among other congenital muscular dystrophy subtypes.

## 2. Patients and Genetic Analysis

### 2.1. Patients

**Figure 1:**
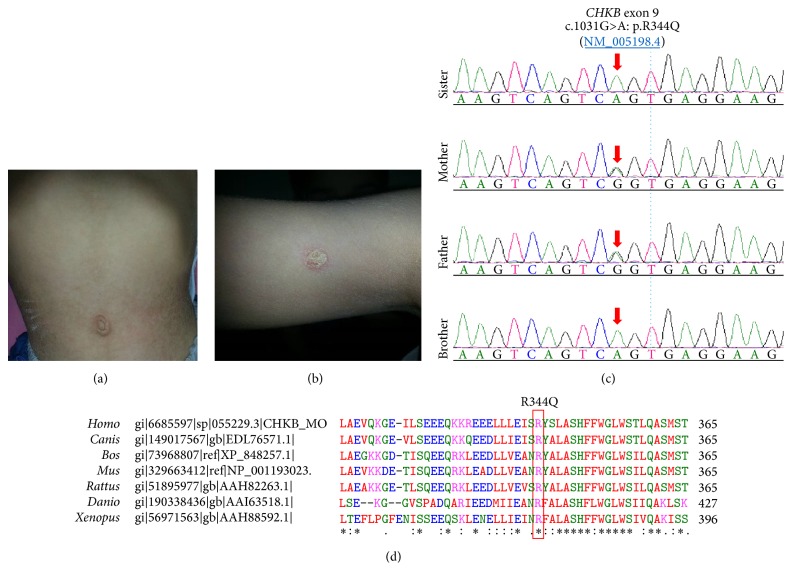
(a) Dry skin with ichthyosis in the younger sibling at the age of 3 years. (b) Dry skin with nummular eczema in the elder sibling at the age of 8 years. (c) Sanger confirmation of the mutation. Both affected siblings are homozygous and both parents are heterozygous carriers for R344Q mutation. (d) The conservation of the homozygous missense mutation within the vertebrates (http://www.ebi.ac.uk/Tools/msa/clustalw2/).

Informed consent was obtained from the parents for genetic investigations, recording and publishing the disease-related information. Two siblings aged three and eight-years-old were admitted for the investigation of psychomotor delay. Both of them were born at term without any complication. The parents were first degree consanguineous. Hypotonia from birth and psychomotor delay were noted. At age of 12 months, they were able to sit without support, but they could never gain the ability to stand and walk. Language delay and fine motor difficulties, which were more prominent in the younger sibling, became evident at the age of 24 months. At the age of 8 years, the elder sibling was able to speak fluently, but the younger sibling had only a few words at the age of 3 years. The elder sibling also learned to read, but was attending to school for special needs, and the Wechsler Intelligence Scale for Children revealed a total intelligence quotient score of 60. They did not have a history of seizures. Physical examination revealed hypoactive deep tendon reflexes, proximal muscle weakness, contractures in the knee of elder sibling, diffuse mild generalized muscle atrophy, dry skin with ichthyosis and multiple nummular eczema in the younger and elder siblings, respectively (Figures 1(a) and 1(b)). Brain and spinal cord magnetic resonance imaging and metabolic investigations including urine and serum amino acids and urine organic acids were normal. Serum creatine kinase values in the younger and elder siblings were 406 IU/L and 520 IU/L, respectively. Nerve conduction studies were normal but needle examination showed myopathic potentials in all examined muscles. Muscle biopsy of the younger sibling at the age of 2.5 years revealed variation in fiber size, increased number of internal nuclei and fatty infiltration. Oxidative enzyme reactions (NADH-TR and SDH) showed enlarged mitochondria at the periphery of most fibers (data not shown). Immunolabelling of *β*-spectrin, dystrophin, dystroglycans (*α* and *β* subunits), calpain, sarcoglycans (*α*, *β*, *γ*, and *δ* subunits), collagen 6, merosin/laminin-2, emerin, myotilin, and laminin A/C was normal (data not shown).

### 2.2. Exome Sequencing

For further genetic analysis, we performed whole exome sequencing (WES) from peripheral blood DNA of the younger male sibling after Nimblegen enrichment (SeqCap EZ Human Exome Library v2.00) on the Illumina HiSeq 2000 platform with 2 × 100 bp according to manufacturer instructions. The coverage was 75-fold, that is, 10x coverage for 96% of target sequences and 30x coverage for 86.2% of target sequences. The Cologne Center for Genomics VARBANK pipeline v.2.12 (https://varbank.ccg.uni-koeln.de/) used for data analysis for rare autosomal recessive disease in a consanguineous family revealed an unpublished novel homozygous missense variant in the Choline Kinase-Beta (*CHKB*) gene c.1031G>A in exon 9 causing p.R344Q according to NM_005198.4 (Figure 1(c)). This variant was not present in the 1000 genomes (http://browser.1000genomes.org/index.html) or Exome Variant Server (http://evs.gs.washington.edu/EVS/) databases, but one allele was detected in 1/66650 European population chromosomes by the Exome Aggregation Consortium (http://exac.broadinstitute.org/). Arginine at 344 is highly conserved on vertebrates (Figure 1(d)) and analysis of this variant predicted it to be disease causing by MutationTaster (http://www.mutationtaster.org). The bioinformatics analysis shows that aberrant amino acid change is located on the choline kinase-motif domain of the protein [5]. The R344Q variant was confirmed by Sanger sequencing, which also revealed the homozygosity for the other affected sibling and carrier heterozygosity for the parents (Figure 1(c)).

## 3. Discussion

Megaconial congenital muscular dystrophy (CMD) is characterized with early-onset hypotonia, muscle wasting, proximal weakness, cardiomyopathy, mildly elevated serum creatine kinase levels, and mild-to-moderate intellectual disability. The disease is caused by the loss-of-function mutations in choline kinase-beta (*CHKB*) [3].

In the review of Mitsuhashi and Nishino [6], ichthyosis-like skin changes have been observed in six patients out of 19. Five of them were of Turkish origin and one of them was of British origin. Ichthyosis-like skin changes were also reported by Quinlivan et al. in two unrelated patients with CMD who were shown to have one frameshift (W284^*∗*^) and one missense (N241S) homozygous mutation in CHKB, respectively [7]. In those cases, ichthyosis in proximal areas with fine scaling was suggested as an important clue to the diagnosis. The largest megaconial CMD patient series by Haliloglu et al. demonstrated that ichthyosis-like changes was the most frequent (11/15) skin change in megaconial CMD [8]. Nummular eczema, which is shown in the elder sister of our patients, has not been described before and the symptoms improved after topical corticosteroid therapy.

Examination of skin is very important in patients with suspected congenital muscular dystrophy. Patients with collagen VI related dystrophies including Ullrich CMD and Bethlem myopathy have keloid or atrophic scar formation, striae, thin skin, soft skin on palms and soles, and hyperkeratosis pilaris [1]. Another severe LGMD type presenting with skin lesions together with congenital muscular dystrophy, myasthenic symptoms, and epidermolysis bullosa simplex is caused by plectin (*PLEC1*) mutations and should be considered in the different diagnosis [9].

## 4. Conclusion

Patients with megaconial congenital muscular dystrophy have ichthyosis-like skin changes with intense pruritus, which will lead to clinical diagnosis in cases with congenital muscular dystrophy. Nummular eczema may also be a new skin finding in megaconial congenital dystrophy, which warrants further reports.
